# “The Worst Part Was Coming Back Home and Feeling Like Crying”: Experiences of Lesbian, Gay, Bisexual and Trans Students in Portuguese Schools

**DOI:** 10.3389/fpsyg.2019.02936

**Published:** 2020-01-15

**Authors:** Jorge Gato, Daniela Leal, Carla Moleiro, Telmo Fernandes, Diogo Nunes, Inês Marinho, Oren Pizmony-Levy, Cody Freeman

**Affiliations:** ^1^Faculty of Psychology and Education Sciences, University of Porto, Porto, Portugal; ^2^Center for Psychology, University of Porto, Porto, Portugal; ^3^ISCTE-University Institute of Lisbon, Lisbon, Portugal; ^4^Teachers College, Columbia University, New York, NY, United States; ^5^Faculty of Learning Sciences and Education, Thammasat University, Bangkok, Thailand

**Keywords:** school climate (or environment), discrimination, bullying, LGBTI, Portugal

## Abstract

Portugal is one of the most egalitarian countries in Europe in terms of lesbian, gay, bisexual, transgender, and intersex (LGBTI) individuals’ legal rights. However, regarding education Portugal still lacks specific policies, plans and interventions to protect LGBTI students. To assess the perceptions of self-identified LGBTI youth regarding their school context, a total of 663 participants (aged from 15 to 20 years old) filled in an on-line questionnaire about their school climate. One hundred and forty-six of them answered an open-ended question about their personal experiences. A thematic analysis of these answers was conducted, and four main categories were identified: (i) victimization, (ii) coming out experiences, (iii) support networks, and (iv) demands. Most participants reported experiences of discrimination, and several sources of prejudice were identified. Furthermore, participants also recognized a lack of LGBTI information in school curriculum and made several demands. Besides inclusive laws, we suggest that the safety and the well-being of LGBTI youths in Portuguese schools depend upon others measures, such as teacher and school staff training, curricula inclusive of LGBTI diversity, and local strategies, such as Gay-Straight Alliances.

## Introduction

School is often a hostile environment for lesbian, gay, bisexual, and transgender (LGBT) youth (Earnshaw et al., 2016; Kosciw et al., 2016; Pizmony-Levy and Kosciw, 2016; Russell and Fish, 2016; Toomey and Russell, 2016; Day et al., 2018; Pizmony-Levy et al., 2019). In fact, bullying based on actual or perceived sexual orientation or gender identity/expression has been identified as a global problem violating sexual and gender minority students’ rights and hindering their educational success (UNESCO, 2012; Pizmony-Levy and Kosciw, 2016).

Research shows that in comparison to their heterosexual and cisgender peers, LGBT youth are more likely to experience victimization, report higher rates of truancy (Birkett et al., 2009; Day et al., 2018), have poorer academic performance (Pearson et al., 2007), report more negative perceptions of school climate (Swearer et al., 2008; Birkett et al., 2009; Day et al., 2018), and experience less sense of belonging to their school (Galliher et al., 2004; Pearson et al., 2007). Besides suffering higher levels of school-based victimization compared to heterosexual and cisgender youth (Toomey and Russell, 2016; Day et al., 2018), LGBT students also have to deal with many tasks and challenges concerning their sexual orientation and gender identity/expression, such as absence of positive role models, lack of coping mechanisms to deal with victimization and self-acceptance, loneliness, and coming out (Savin-Williams, 1998).

Given that higher levels of social stigmatization are associated with psychological distress (Meyer, 2003), sexual and gender minority youth are at risk for a variety of poor health and well-being related outcomes (Marshal et al., 2011; Rosario and Schrimshaw, 2013; Olson-Kennedy, 2016; Espelage et al., 2018; Hatchel et al., 2018). More specifically, discrimination and victimization in schools are associated with higher levels of depression, self-harm, and suicidal ideation in this population (Almeida et al., 2009; Shields et al., 2012; UNESCO, 2012; Perez-Brumer et al., 2017; Day et al., 2018; Espelage et al., 2018).

Given this state of affairs, research highlighted the importance of understanding the specific challenges LGBT youth face in school and improving school climate to assure their well-being (Russell et al., 2011; Russell and Fish, 2016). In Portugal, where the present study was conducted, there is a lack of national level data collection of bullying and harassment toward this population in schools (Lgbtqi Inclusive Education Report, 2018). Furthermore, research about the experiences of LGBT students is scarce (for exceptions see António et al., 2012; Freitas et al., 2015; Rodrigues et al., 2016; Santos et al., 2017, 2018). Thus, our goal in this work was to analyze the written comments left by a group of LGBT students who answered an online questionnaire about their school experiences in Portugal.

The above-mentioned challenges are not restricted to the school environment since LGBT adolescents might be at the same time in vulnerable positions in different contexts. Next, we will review what is known about the experiences of LGBT adolescents in school and in their relations with friends and family. Because the surrounding social and legal climate play a major role in the lives of sexual and gender minority individuals (Bauermeister, 2014), we will report next some specificities of the Portuguese context, as well as what studies conducted in this country with LGBT adolescents have revealed so far.

### Portuguese Context

Portugal is a southern European country, with a catholic cultural matrix and a 45-year-old democracy, struggling to rise to the economic and educational development challenges posed by its European Union membership status. With little more than two decades of active lesbian, gay, bisexual, transgender, and intersex (LGBTI) movement, the last 15 years brought nevertheless a significant amount of changes in the legal and social contexts regarding sexual and gender diversity acknowledgment, awareness and respect.

Some legal landmarks are the inclusion of sexual orientation in the principle of equality and non-discrimination of the Constitution of the Portuguese Republic (2005), the marriage equality law in 2010 (Law no. 9/2010, 2010 Diário da República), a first gender identity law in 2011 (Law no. 7/2011, 2011 Diário da República), subsequently upgraded in 2018 to include self-determination of trans people and the possibility to change legal gender at the age of 16 (Law no. 38/2018, 2018 Diário da República), and equal access to adoption for same sex couples in 2016 (Law no. 2/2016, 2016 Diário da República). A bill on sex education includes, since 2009, sexual orientation and gender identity in its provisions (Law no. 60/2009, 2009), and the law on the status of the student and school ethics (Law no. 51/2012, 2012 Diário da República) offers protection on the grounds of sexual orientation and gender identity for gender and sexual minority youth. The rights of intersex people have finally been acknowledged in the National Strategy for Equality and Non-Discrimination, approved in 2018 (Resolution of the Council of Ministers no. 61/2018, 2018 Diário da República).

However, there is no assessment to what is actually being done to bring about change in school context, and the absence of a specific strategy is evident. At least two NGO’s (ILGA Portugal and *rede ex aequo*) have been promoting awareness raising activities in schools on a national level, including storytelling, peer meetings and, since 2017, students’ alliances (ILGA Portugal, 2019). But resources are scarce and there is little evidence about the effectiveness of these strategies at a local level.

Research about the experiences of LGBTI students is also scarce in Portugal. However, a previous work about homophobic bullying in Portuguese schools (António et al., 2012) revealed that psychological violence and victimization among LGBTI identified students are more prevalent in boys; that aggressive behaviors toward LGBTI students are generally undervalued; that witnessed situations lack intervention; and that there are significant psychological consequences for the victims of homophobic bullying. Consistently, Rodrigues et al. (2016) showed that Portuguese LGBTI adolescents reported several types of discrimination and bullying against them: masculine violence, feminine violence, violence with less perceived impact, and violence with greater perceived impact. Furthermore, it seemed that most victims of homophobic bullying were afraid of revealing their sexual orientation to their family and they did not report homophobic episodes in school to their family. Santos et al. (2017, 2018) explored Portuguese high school students’ opinions about sexual diversity and verified that their discourses gravitated between liberal acceptance, conditional acceptance and intolerance. Furthermore, the expression of homophobia in schools was strongly related to processes of masculinity construction. The authors drew attention not only to discrimination against LGBT youth but also to the gap between what is legally decreed and the lack of effective approaches to tackle sexual diversity issues in schools. Regarding family experiences of Portuguese LGBT youth, while Freitas et al. (2015) found that having frequent experiences of conflict with parents exacerbated the negative impact of discrimination episodes on adolescents’ mental health, António and Moleiro (2015) verified that parental support was a moderator of the effects of homophobic bullying on psychological distress. More recently, the National Survey on School Climate conducted in Portugal in 2016/2017 (Pizmony-Levy et al., 2018) revealed that schools are for many LGBTI youngsters an environment of unsafety and discomfort, in which insults and other negative attitudes are frequent. In this regard, 37% of inquired students reported they felt unsafe because of their sexual orientation and 28% because of their gender expression. Approximately one in four students said he/she avoided using spaces such as showers, bathrooms or sports classes, due to feelings of unsafety and discomfort. Areas such as the school cafeteria were also avoided. At least one out of six students missed classes because he/she felt unsafe or uncomfortable. In the present work, we aimed at preserving the authentic voice of youth as they reflect on their experience through the analysis of open-ended comments reported by participants of the afore-mentioned National Survey on School Climate (Pizmony-Levy et al., 2018).

### LGBT Youth in School

School is potentially a difficult environment for LGBT students. Homophobic and transphobic bullying include teasing, name calling and public ridicule, spreading rumors about one’s sexual orientation or gender identity (also known as outing), intimidation, pushing and hitting, stealing or damaging belongings, social isolation, cyber bullying (harassment through email, cell phones, text messages, defamatory websites, and social media), physical or sexual assault, and death threats (UNESCO, 2012). This type of bullying is often perpetrated by students, but in some cases by teachers and other school staff (UNESCO, 2012). In this regard, Pizmony-Levy et al. (2008) found out that nearly half of their sample of LGBT students in Israel, which ranged in ages from 11 to 18 years, reported occasionally hearing homophobic remarks uttered by most of the teachers. Besides the perpetrator and the victim, bullying also involves and affects other persons, including those who witness or are bystanders. Worryingly, it seems that most of the assaults are witnessed but there is no intervention to stop them (e.g., António et al., 2012). An aspect unique to transgender students, relates to school uniform policies and sanitation facilities that are still gender binary in most educational settings (UNESCO, 2012; Kosciw et al., 2016).

As mentioned before, LGBT students are more vulnerable to bullying than their heterosexual and cisgender peers. Potential reasons for this greater likelihood of abuse include disclosing sexual minority status to others (D’Augelli et al., 2002), behaving in a gender-nonconforming way (D’Augelli et al., 2002; Friedman et al., 2006), or being perceived to be LGBT even when they are heterosexuals and/or cisgender (Swearer et al., 2008; Rodrigues et al., 2016). Boys are the main victims of homophobic bullying from earlier ages when compared to girls (António et al., 2012; UNESCO, 2012; António and Moleiro, 2015; Rodrigues et al., 2016) and negative attitudes and behaviors toward sexual minorities and gender non-conforming persons are more prevalent among male than female students (Kimmel and Aronson, 2003; Costa and Davies, 2012; UNESCO, 2012; Santos et al., 2017).

However, research also shows that LGBT youth fare better (e.g., feel safer, hear fewer homophobic remarks, have more academic success) in schools with programs that address their needs and concerns (e.g., Gay-Straight Alliances) (Russell et al., 2009; Poteat et al., 2013; Toomey and Russell, 2013; Ioverno et al., 2016; Marx and Kettrey, 2016; Day et al., 2019) and with teachers and classmates who are supportive (Kosciw et al., 2013, 2014). LGBT-inclusive curricula were also found to be associated with higher reports of safety at the individual and school levels (Snapp et al., 2015; Gegenfurtner and Gebhardt, 2017). Regarding this aspect, the Lgbtqi Inclusive Education Report (2018) indicated that in Portugal there is a lack of specific policies and action plans, inclusive national curricula, and mandatory teacher training regarding LGBTI issues.

### LGBT Youth and Their Peers

Peer groups constitute one the most important sources of social support during adolescence (Carr, 1999). In fact, LGBT individuals seem to rely more heavily on “chosen families” and friends for everyday social support than on their own families (Frost et al., 2016).

Although sexual minority youths do not differ from their heterosexual peers in the number of friends and the frequency of contact, or level of emotional closeness with these friends (Diamond and Lucas, 2004; Beals and Peplau, 2006; Ueno et al., 2009), they are more likely to report having lost friends and to worry about losing friends (Diamond and Lucas, 2004). Furthermore, losing friends and the intense fear of losing more friends have been associated with psychological distress among LGB youth (D’Augelli, 2002; Diamond and Lucas, 2004). Despite these fears, most LGB youth disclose their sexual orientation to a heterosexual friend in the first place, and almost all have disclosed to a friend (Beals and Peplau, 2006; Rosario et al., 2009). In this regard, the quality of youths’ relations with friends seems to be unaffected by the disclosure of sexual orientation (Beals and Peplau, 2006). The support and acceptance from friends acts as a buffer and is protective for the mental health and well-being of LGB youth (for a review see Rosario and Schrimshaw, 2013) and has been found to be just as beneficial as support from family (e.g., Vincke and Van Heeringen, 2002; Ueno, 2005; Sheets and Mohr, 2009; Rosario et al., 2011).

### LGBT Youth and Their Family

Families often reflect the existing wider societal stigma and become a source of discrimination for LGBT youth (Savin-Williams and Ream, 2003; D’Augelli et al., 2005; Ryan et al., 2009; Rodrigues et al., 2016), increasing mental distress not only during adolescence but also into young adulthood (Freitas et al., 2015; McConnell et al., 2016).

For this reason, many LGBT youth may be reluctant to disclose their developing sexual orientation to their families for fear of negative reactions, resulting in increased difficulties in family relationships. As an example, in the United States only one-third of the LGB youth had experienced positive reactions when they revealed their sexual orientation to their parents; the other one-third experienced negative reactions, with the remaining one-third not disclosing to one or both parents even by their late teens and early 20s (D’Augelli, 2002; Savin-Williams and Ream, 2003; Rosario et al., 2009). Negative reactions from parents typically consist of denial, silence, distancing, and avoidance (Savin-Williams and Ream, 2003), with verbal or physical abuse also occurring (Savin-Williams and Ream, 2003). After the initial reactions, parents tend to become slightly more open to their child’s sexual identity over time (Vincke and Van Heeringen, 2002; Savin-Williams and Ream, 2003; Beals and Peplau, 2006).

Low levels of family support are associated with negative mental health outcomes (e.g., Rosario et al., 2009; Ryan et al., 2009; Needham and Austin, 2010). Furthermore, low levels of family support are related with lower levels of sexual identity integration among LGB youth, including less disclosure, more internalized homophobia, lower involvement in LGBT communities (Rosario et al., 2008), and lower levels of school belonging (Watson et al., 2016).

Nevertheless, families may also play a protective role in the positive and healthy development of LGBT youth (Doty et al., 2010; Rothman et al., 2012; António and Moleiro, 2015; Watson et al., 2019). In this regard, sexual minority youth who felt supported by their parents also showed lower levels of depression and a more positive self-esteem (Watson et al., 2019).

In sum, difficulties experienced by LGBT youth in different contexts, such as school, friends and family, undermine their mental, behavioral, and physical health (Rosario and Schrimshaw, 2013; Earnshaw et al., 2016). In order to conceptualize interventions that assure the improvement of Portuguese LGBT youths’ living conditions and well-being (Lgbtqi Inclusive Education Report, 2018), it is thus important to gather information about their school climate, personal experiences, and social resources.

## Materials and Methods

### Participants

Data were drawn from a larger study with a total of 663 LGBTI students who filled in an online questionnaire about their experiences in school during the last school year. At the end of this survey, there was an open-ended question, in which we asked participants to report anything they considered important about their school experiences as LGBTI students. Our final sample was thus composed of 146 participants who had answered this open-ended question. This is a convenience sample which ranged from 15 to 20 years of age (*M* = 17.01; *SD* = 1.42). Regarding sex assigned at birth, most participants were female (*n* = 94). Concerning gender, 54.1% identified themselves as female (*n* = 79); 38.6% as male (*n* = 49); 7.8% as transgender (*n* = 11); and 5.5% as non-binary (*n* = 8). In terms of sexual orientation, 34 persons defined themselves as gay, 33 as lesbians, 42 as bisexuals (of which 31 defined themselves as females and 10 as males), 17 as pansexuals, seven as queer, six as heterosexuals, three as questioning, two as demisexuals, and two as asexuals. Concerning gender identity, most participants were cisgender (81.5%; *n* = 119). Regarding educational level, the large majority were attending high school (80.4%; *n* = 117), 16.8% had attended between seven and nine years of school and 2.8% had finished high school. Finally, 89% of our participants were born in Portugal. Regarding ethnicity, 87% of the sample was Caucasian/European/White.

### Procedure and Measures

Data were collected online between June and August 2017. Youth were eligible to participate in the study if they were between the ages of 14 and 20, attended school in Portugal during the 2016–2017 school year, and identified themselves as lesbian, gay, bisexual, or a sexual orientation other than heterosexual; or described themselves as transgender or as having another gender identity other than cisgender. The survey instrument was modeled after GLSEN’s 2015 National School Climate Survey and was adapted to the Portuguese context, by the Portuguese authors of this paper. To obtain a large and diverse sample of LGBTI youth in Portugal, the study was advertised and promoted on social media, such as Facebook, Instagram, and Twitter with the following information: “[Are you a] LGBTI youngster? Your voice matters. Go to enae.ilga-portugal. pt and share your school experiences. To participate you must be +14 years; identify yourself as lesbian, gay, bisexual, trans, intersexual or other non-normative identity; and attend high school or an equivalent education pathway in Portugal.”

Participants were informed about the anonymous nature of the study reporting their consent (or not) to participate. Consenting participants were redirected to the online survey. They completed self-report measures about their school experience (e.g., the frequency of discrimination episodes) and at the end of the questionnaire there was an open ended question: “Is there anything else you would like to share about your school experience? Remember this survey is anonymous so, you do not have to write your name, e-mail or another personal data. Furthermore, we are not able to answer your questions or comments in this webpage. If you need to contact us or another LGBTI support service, please consult the information at the last page of this questionnaire.” There was not a word limit in that section and no monetary reward was offered to participants. The confidentiality of these answers was ensured with the survey link being accessed via secure university services in Portugal.

Then, a thematic analysis was conducted (Bold, 2012; Bardin, 2015) to explore participants’ answers to this question.

### Data Analysis

**FIGURE 1:**
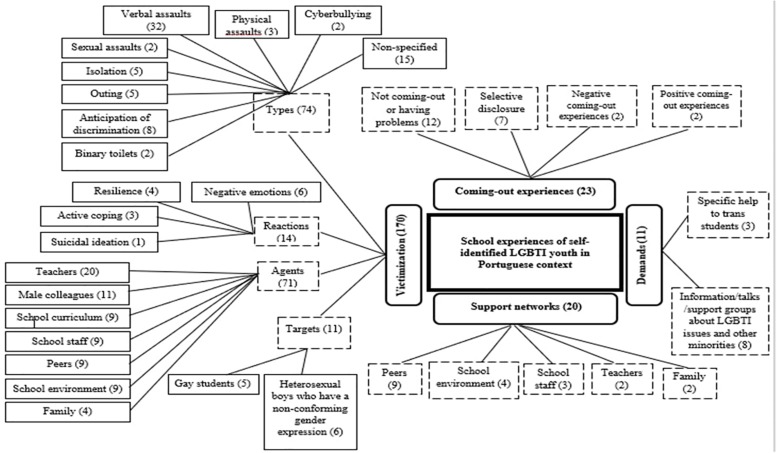
Thematic map: categories, subcategories and number of occurrences.

We conducted our thematic analysis through a descriptive perspective to categorize common themes emerging from the participants’ answers (Bold, 2012; Bardin, 2015). First, we read carefully all the participants’ answers and a preliminary draft of the categorical system was created. Then, we systematically read the material to identify the main categories and sub-categories, reflecting common underlying issues, and the number of occurrences. In Figure 1, we reported the main categories; sub-categories and the number of occurrences.

Two quality control steps were used to provide the validation of the analysis. To validate the final categorization, the authors met for 2 h to discuss and validate the categories (Yardley, 2008). Furthermore, two undergraduate research volunteers blindly rated all categories. The agreement between the two independent coders was 95%.

## Analysis and Discussion of Results

We identified four main categories: Victimization, Coming out experiences, Support networks, and Demands (see Figure 1).

Victimization related contents were the most mentioned (170 occurrences), distributed across four second-level categories. This way, participants provided information about the types of victimization they suffered or witnessed (74 occurrences), the perpetrators or agents of victimization (71 occurrences), their reactions (14 occurrences) and, finally, who were the main targets of these events (11 occurrences). The vastness of this theme is consistent with what studies have found in other countries, that is, school is often an unfriendly place for LGBT youth (UNESCO, 2012; Earnshaw et al., 2016; Kosciw et al., 2016; Pizmony-Levy and Kosciw, 2016; Russell and Fish, 2016; Toomey and Russell, 2016; Day et al., 2018; Pizmony-Levy et al., 2019).

Occurrences were distributed evenly regarding participants’ Coming-out experiences (23) and Social support networks (20). Coming-out is one of the many tasks and challenges LGBT youths have to deal with (Savin-Williams, 1998). Given reported experiences of victimization it is not surprising that participants mentioned mostly that they had not come out or had problems during this stage (12 occurrences), followed by selective self-disclosure experiences (seven occurrences), overall negative experiences (two occurrences), and only two positive coming-out accounts.

As Social support networks, students identified mostly their peers (nine occurrences), followed by the school environment (four occurrences), school staff (three occurrences), teachers and family (both with two occurrences each). It is interesting to note that all sources of social support were also mentioned as agents of victimization. However, with the exception of peers, these instances – teachers, school environment, school staff, and family – were characterized more frequently as agents of victimization than as support networks. For instance, teachers were mentioned 20 times as agents of victimization but only two times as sources of support.

Finally, participants made some Demands (11 occurrences), related mainly with information needs about LGBTI issues (eight occurrences) and, more residually, regarding specific help to trans students (three occurrences).

Next, we will describe, analyze and discuss each one of the categories in further detail, to characterize quotations of each participant we will use gender, age, sexual orientation, and gender identity.

### Victimization

This category included four second-level subcategories: (i) types of victimization, (ii) agents of victimization, (iii) reactions to victimization, and (iv) targets of victimization.

#### Types of Victimization

Regarding the first subcategory, nine forms of **Victimization** were identified, including non-specified bullying/discrimination, cyberbullying, physical assaults, verbal assaults, sexual assaults, isolation, outing, anticipation of discrimination, and binary toilets. Interestingly, these categories correspond to the definition of homophobic and transphobic bullying proposed by the UNESCO (2012). Consistently with previous research, reports of psychological violence were more frequent than reports of physical violence (António et al., 2012; António and Moleiro, 2015; Earnshaw et al., 2016; Rodrigues et al., 2016). Thus, the most represented subtheme related to **Verbal assaults:** “During recess and if I was alone it was common to be called gay” (Male, 19, gay, cisgender); followed by **Non-specified bullying/discrimination**: “…in my last five years of school (…) I was a constant victim of bullying…” (Male, 16, gay, cisgender); **Anticipation of discrimination:** “If I come-out as gay to everybody, probably I would have to hear some insults as fag (…)” (Male, 15, gay, cisgender); **Isolation:** “There was a lot of prejudice, I lost all my «friends» [brackets in the original statement], my class put me aside completely…” (Female, 19, lesbian, cisgender); and **Outing:** “Without my permission, a school janitor (till this day I don’t know who) informed my parents about my alleged sexual orientation.” (Female, 17, pansexual, cisgender). The least mentioned subthemes related to **physical violence**, namely physical assaults: “My colleagues used to strangle my neck in 7th grade because I was gay” (Male, 16, gay, cisgender); and **sexual assaults:** “I was in a bench talking with my girlfriend and a slightly younger boy approached us and stood still one meter away from us with the hand inside his pants, as if he was touching himself” (Female, 17, bisexual, cisgender). **Cyberbullying**, a more recent form of harassment was residually mentioned: “A boy was extremely made fun of because nudes and intimate videos of him were spread” (Female, 17, lesbian, cisgender). Finally, **binary toilets** not inclusive of trans and non-binary identified students were also mentioned as a form of victimization.

#### Agents of Victimization

Concerning the sub-category Agents of victimization, **Teachers** emerged as the most frequent perpetrators of discrimination: “My Portuguese language teacher [male] was a homophobe and was constantly making [homophobic] remarks” (Female, 18, questioning, cisgender). Already highlighted in previous studies (Pizmony-Levy et al., 2008; UNESCO, 2012), this is a worrying result, not only because teachers are important role models for students but also for ethical reasons. In fact, the IGLYO report (2018) reported a lack of mandatory teacher training regarding LGBTI issues in Portugal, and this seems to be consistent with participants’ perceptions. **Male colleagues** were the second most frequent cited perpetrators of bullying: “My experiences in school were terrible because it was a men’s college only” (Male, 20, gay, cisgender). This result is in accordance with the fact that males have more negative attitudes toward sexual minority’ individuals than their female counterparts (LaMar and Kite, 1998; Kimmel and Aronson, 2003; UNESCO, 2012), including among high school students (Costa and Davies, 2012; Santos et al., 2017). Again, consistently with the results and recommendations of the Lgbtqi Inclusive Education Report (2018) regarding the absence of specific policies and action plans and inclusive national curricula concerning LGBTI issues in Portugal, **School curriculum** appeared as a frequently mentioned source of prejudice and discrimination: “(…) Talking about LGBT issues is really rare. Since my 1st year at school until now, at high school, I never had a lecture or a class regarding LGBT questions (…)” (Male, 16, gay, cisgender). Regarding **School staff**, one participant mentioned that “a school employee talked about our relationship to my girlfriends’ family and ruined our relationship” (Female, 16, lesbian, cisgender). This particular result suggests that mandatory training regarding LGBTI issues recommended by the Lgbtqi Inclusive Education Report (2018) should include not only teachers but also other school workers. **Peers** in general were also mentioned as perpetrators of bullying: “In my school, with my colleagues, it’s the only place where I don’t feel comfortable revealing my sexuality” (Female, 15, pansexual, cisgender). **School environment** was mentioned as many times as School curriculum, School staff and Peers: “The school doesn’t have a very healthy environment in terms of diversity” (Male, 15, gay, cisgender) as agent of victimization, which is both consistent with international scholarship (Galliher et al., 2004; Pearson et al., 2007; Swearer et al., 2008; Birkett et al., 2009; Kosciw et al., 2013, 2014; Snapp et al., 2015; Gegenfurtner and Gebhardt, 2017; Day et al., 2018) and the situation of LGBT youths in Portuguese schools (Lgbtqi Inclusive Education Report, 2018).

Interestingly, **Family** was the least cited source of prejudice: “My parents don’t allow me to tell others I’m trans” (male, 17, heterosexual, trans man). Although families often reproduce the wider social stigma against LGBTI individuals (Savin-Williams and Ream, 2003; D’Augelli et al., 2005; Ryan et al., 2009; Rodrigues et al., 2016) with long-term consequences for LGBTI youth’s mental health (Freitas et al., 2015; McConnell et al., 2016) it may be that, as Rodrigues et al. (2016) reported because LGBTI youngsters tend not to reveal their sexual orientation to their parents and siblings in a first phase, family is not seen as an active agent of victimization.

#### Reactions to Victimization

Regarding the way participants react to prejudice and discrimination, **Negative emotions** (e.g., fear, sadness, shame, etc.) were the most prevalent reaction. As one participant wrote, “The worst part was coming back home and feeling like crying” (Male, 19, gay, cisgender). This is an expected result, as stigmatization is associated with distress (Meyer, 2003) putting youngsters at risk for poorer general well-being and mental health (Savin-Williams, 1998; Marshal et al., 2011; Rosario and Schrimshaw, 2013; Olson-Kennedy, 2016; Espelage et al., 2018; Hatchel et al., 2018).

Despite being cited only four times, **Resilience** was the second most prevalent reaction to victimization: “Although the extremely negative remarks in school, and outside school, were almost daily, I never stopped wearing my clothes and I intend to keep thinking the same thing about this issue” (Female, 18, questioning, cisgender). Closely related to Resilience, **Active coping** was mentioned in three occasions, “I reported persons to the school principal twice because of violation of school ethics (verbal aggressions/lack of respect)” (Male 17, heterosexual, trans man). A closer look at these two last subcategories reveals that both relate to individual attitudes, behaviors or characteristics such as self-esteem and resisting cisheteronormativity. This result suggests that among our participants resiliency depends more upon personal characteristics than contextual and social protective factors (Masten, 2016; Freitas et al., 2017), such as a safe school environment or social support. In fact, in Portugal schools where the issues of sexual orientation and gender identity are addressed seem to be the exception and not the rule (Lgbtqi Inclusive Education Report, 2018). Although victimization is associated with a greater incidence of depression, self-harm and suicidal ideation (Almeida et al., 2009; Shields et al., 2012; UNESCO, 2012; Perez-Brumer et al., 2017; Day et al., 2018; Espelage et al., 2018; Hatchel et al., 2018), the subcategory **Suicidal ideation** was only mentioned once.

#### Targets of Victimization

Consistently with previous investigation (Kimmel and Aronson, 2003; António et al., 2012; Costa and Davies, 2012; António and Moleiro, 2015; Rodrigues et al., 2016), our results indicate that discrimination operates in function of sexual orientation and gender expression, with boys being in both cases the main targets of discrimination. Regarding **Gay students**, one participant mentioned that “I mainly notice that it is gay and bi[sexual] boys who are discriminated” (Female, 18, bisexual, cisgender). Concerning **heterosexual boys who have a non-conforming gender expression**, they are often perceived as homosexual or bisexual (Rodrigues et al., 2016) and are victims of prejudice and discrimination: “Remarks such as «queer» and «girlie» are still used to describe slightly effeminate behaviors of straight boys” (Female, 16, bisexual, cisgender). In fact, boys seem to be the main victims of homophobic bullying (António et al., 2012; António and Moleiro, 2015) since earlier ages than girls (Rodrigues et al., 2016).

### Coming-Out Experiences

Unsurprisingly, **Not coming out or having trouble in this process** was the most mentioned experience (D’Augelli, 2002; Savin-Williams and Ream, 2003; Rosario et al., 2009). As one participant said, “Many people, like me, do not feel safe to come-out in school” (Female, 18, lesbian, cisgender). Youths fear losing their social support networks if they disclose their sexual and gender minority status (D’Augelli, 2002; Diamond and Lucas, 2004) and, in fact, it has been found that this disclosure renders them more vulnerable to bullying (D’Augelli et al., 2002). A strategy is **Selective disclosure**, that is, revealing one’s sexual orientation to a group of persons who are identified as allies, “Only one friend knows that I am bisexual because I do not feel comfortable to come-out to everybody” (Male, 17, bisexual, cisgender). Two **Negative coming-out experiences** were also described, “I came-out of the closet to my class and to my teacher and few people took it well (…)” (Male, 17, demisexual, transgender man). However, **Positive coming-out experiences** were also mentioned, “I gave a talk about transsexuality during which I came out as transsexual. In the end of this talk everybody was in tears, waiting in line to give me a hug and give me strength, it was awesome!.” (Male, 17, heterosexual, transgender man). In fact, coming-out does not have to be necessarily associated with pain and lack of self-acceptance as most identity development models predict and more knowledge about positive coming-out experiences is needed (Clarke et al., 2010).

### Support Networks

Consistently with literature (Carr, 1999; Beals and Peplau, 2006; Rosario et al., 2009; Ueno et al., 2009; Frost et al., 2016), **Peers** were the most mentioned source of social support, “My friends accepted me as I am” (Female, 18, bisexual, cisgender). The **School environment** was qualified four times as supportive, “I think my school and its students are quite open about the LGBT community” (Female, 15, questioning, cisgender). Although not frequently, **Teachers** and **School staff** members were also perceived by some participants as supportive, “(…) a colleague made a homophobic comment and the teacher countered him (…)” (Female, 17, lesbian, cisgender). Again, more knowledge needs to be gathered about LGBT youths’ positive experiences. In this regard, literature has already shown that merely having anti-discrimination policies does not confer significant protection to LGBT youths (Chesir-Teran and Hughes, 2009). What seems to make a difference are classroom discussions, books in the school library that address sexual and gender diversity (Goodenow et al., 2006; Chesir-Teran and Hughes, 2009), support from teachers and classmates (Kosciw et al., 2013, 2014), and specific support programs, such as Gay-Straight Alliances (Russell et al., 2009; Poteat et al., 2013; Toomey and Russell, 2013; Ioverno et al., 2016; Marx and Kettrey, 2016; Day et al., 2019).

Even though parents tend to be more accepting of their child’s sexual identity over time (Vincke and Van Heeringen, 2002; Savin-Williams and Ream, 2003; Beals and Peplau, 2006) and **Family** can be protective factor of LGBT youths well-being (Doty et al., 2010; Rothman et al., 2012; Watson et al., 2019), this network was only mentioned twice as a source of social support. This low frequency may be related to the fact that our participants are still in the process of revealing their sexual orientation and/or gender identity to their family, experiencing mostly difficulties in this stage (Savin-Williams and Ream, 2003; D’Augelli et al., 2005; Rosario et al., 2008, 2009; Ryan et al., 2009; Needham and Austin, 2010; Freitas et al., 2015; McConnell et al., 2016; Rodrigues et al., 2016; Watson et al., 2016). Finally, **LGBTI groups/peers** were also identified twice as a support network, “I eventually found out that some [female] friends of mine were LGBT and together we ended up meeting other persons, all from school, this way I didn’t feel alone and I was not afraid anymore” (Female, 17, lesbian, cisgender).

### Demands

Considering all challenges to LGBTI students, some demands emerged from their discourses. These demands included (i) specific help to trans students and (ii) information/talks/support groups about LGBTI issues and other minorities. Regarding specific help to trans students “I wish there was someone in my school, or in my city, who was expert in LGBT + questions, to talk to me or to someone who needed, and to help, for example, in the process of sex transitioning” (Female, 17, pansexual, trans woman). In turn, similar demands were asked considering all LGBTI community and other minorities “(…) the school should have support groups to help the inclusion of the LGBTI community. I hope this will happen in the future” (Female, 16, pansexual, cisgender). Students’ perceptions are consistent with the lack of inclusive school programs and training of teachers and school staff (Lgbtqi Inclusive Education Report, 2018).

### Concluding Remarks

Perceptions of our participants concerning victimization processes, coming out experiences, support networks, and demands are consistent with the evaluation and recommendations of the Lgbtqi Inclusive Education Report (2018) regarding Portugal. In brief, our findings point to (i) the urgency of mandatory teacher and school staff training regarding LGBTI issues, (ii) the adoption of specific policies, action plans, and inclusive national curricula concerning LGBTI issues, and (iii) the implementation of programs that address LGBTI youths’ needs and concerns, such as Gay-Straight Alliances. In this regard, it is worth mentioning the pioneering project “Alianças da Diversidade,” implemented by ILGA-Portugal whose aim was to foster Gay-Straight Alliances in the Northern Region of Portugal (ILGA Portugal, 2019).

Despite its informative and potentially useful results, this study is not without its limitations. First, written comments only provide a glimpse of students’ subjective experiences and future research should consider in-depth interviews as a data collection method to extend the assessment of their experiences and perspectives. Second, the study would gain in representativeness with the inclusion of quantitative data about the experiences of the inquired sample. This way, our results would benefit from being confronted in future works with more general tendencies identified by the National Survey on School Climate (Pizmony-Levy et al., 2018). Finally, cultural comparisons might also shed light on the importance of contextual factors on students’ well-being.

Nevertheless, taking into account the scarcity of national data, the present study constitutes an important step forward in knowing more about Portuguese LGBTI youths’ experiences in schools and with their peers and family.

## Data Availability Statement

The datasets generated for this study are available on request to the corresponding author.

## Ethics Statement

This study was reviewed and approved by the Institutional Review Board at Teachers College, Columbia University, a waiver for parental consent was provided by this ethics committee.

## Author Contributions

All authors made substantial intellectual contributions to the work, revised the manuscript, and approved it for publication. OP-L and CF designed the study and managed the data file. JG, DL, CM, DN, and TF adapted the questionnaire to Portuguese language and implemented the on-line survey in Portugal. DL, JG, CM, and IM developed the thematic coding scheme and coded the responses. JG took the lead in writing the manuscript.

## Conflict of Interest

The authors declare that the research was conducted in the absence of any commercial or financial relationships that could be construed as a potential conflict of interest.
